# The mid-pachytene transition between early and late double-strand breaks in *C. elegans* meiosis is conserved throughout early adulthood and coincides with a peak in recombination intermediates

**DOI:** 10.1093/g3journal/jkag130

**Published:** 2026-05-14

**Authors:** Hui Tian, Korie Campbell, Sarit Smolikove

**Affiliations:** Department of Biology, The University of Iowa, Iowa City, IA 52242, United States; Department of Biology, The University of Iowa, Iowa City, IA 52242, United States; Department of Biology, The University of Iowa, Iowa City, IA 52242, United States

**Keywords:** *C. elegans*, meiosis, DSB, crossover, WormBase

## Abstract

In meiosis, SPO-11-induced DNA double-strand breaks (DSBs) are repaired via 2 main homologous recombination pathways: the double Holliday Junction pathway, which yields crossovers (COs), and the synthesis-dependent strand annealing pathway, which generates non-crossovers (NCOs). The standard model assumes that the choice between these 2 outcomes occurs simultaneously and late in the pathway, following strand invasion. We previously demonstrated that in *Caenorhabditis elegans*, this decision is made early: DSBs formed early in meiotic prophase are destined for NCOs, while late DSBs are committed to COs. In this study, we aimed to identify if and how the time window for early-to-late DSB transition is altered when the germline grows. It is established that the germline grows as a worm develops through early adulthood. We find that the expansion of the pachytene stage of meiotic prophase I is the main driver for germline growth. We further characterize the movement of meiotic nuclei and identify that while the average movement rate is consistent with previous studies, surprisingly, nuclear movement gradually slows during meiotic prophase I. Furthermore, the late DSB (CO-fated) region expands proportionally with the growing pachytene, consistently corresponding to the proximal half of the pachytene region. These findings indicate that the early-to-late DSB transition coincides with the peak of RAD-51 foci numbers, which remains stably positioned at the middle of pachytene during germline growth. Thus, our data not only provide a precise measurement of early adult germline development but also suggest that the time window for early-to-late DSB transition coincides with mid-pachytene signaling.

## Graphical Abstract

**Graphical Abstract:**
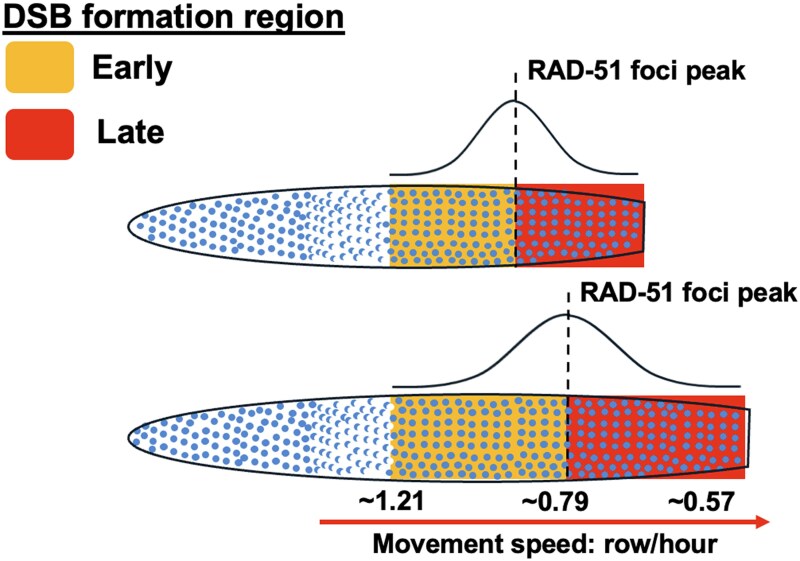

## Introduction

Meiosis is the process of producing gametes, which contain half the genetic complement of the parental cell. To do so, cells execute 2 divisions in tandem, the first of which is unique to meiosis as it involves the segregation of homologous chromosomes (Zickler and Kleckner 2023). To accurately separate chromosomes during the first meiotic division, homologous chromosomes are connected by a mechanism that requires the exchange of DNA segments, which generates crossovers (COs) (Payero and Alani 2025). Formation of at least 1 CO per chromosome pair is essential for meiosis, as chromosomes that fail to do so segregate their chromosomes randomly, frequently leading to inviable gametes (Zickler and Kleckner 2023).

CO formation requires the generation of programmed DNA double-strand breaks (DSBs), which serve as a substrate for homologous recombination (HR) (Payero and Alani 2025). However, not all DSBs formed in meiosis generate COs, the reciprocal exchange of DNA between homologous chromosomes. In fact, most DSBs are processed to form non-COs (NCOs), in which some DNA sequences are copied from the homologous chromosome without exchange (McMahill et al. 2007). Although both COs and NCOs are products of HR, in meiosis, 2 distinct HR sub-pathways generate these diverse outcomes: double Holliday Junction (dHJ) pathway (also called DSB repair) and synthesis-dependent strand annealing (SDSA) (Baudat and de Massy 2007; Andersen and Sekelsky 2010). While the dHJ pathway forms predominantly COs, SDSA results in NCOs. In mitosis, dHJ can be processed by dissolution, which leads to NCO formation (Bizard and Hickson 2014). In meiosis, however, this pathway is minor, and most NCOs are a result of SDSA (Allers and Lichten 2001; McMahill et al. 2007; Youds and Boulton 2011). The mechanisms that govern where and when each pathway is used are still under investigation.

Since DSBs are essential for CO formation, meiosis cannot rely on random DNA damage, so meiotic DSBs are intentionally formed. These programmed DSBs are induced by the topoisomerase-like protein, Spo11, during meiotic prophase I (Bergerat et al. 1997; Keeney et al. 1997). The formation of DSBs during meiosis is a strictly regulated process that commits their repair to the HR pathway (Murakami and Keeney 2008). HR repair is initiated with DSB end resection, the process of generating single-stranded DNA (ssDNA) from double-stranded DNA (dsDNA) by digesting one strand of the dsDNA molecule. Meiotic resection is required for HR since it generates the 3′ ssDNA overhang that is used for strand invasion and the replication of DNA sequences from the homologous chromosomes (Gnügge and Symington 2021). Spo11 covalently binds to the DNA during DSB formation (Keeney et al. 1997). Therefore, removal of Spo11 is required before resection can proceed (Stracker and Petrini 2011; Cejka and Symington 2021). This action links meiotic DSB formation to resection, and therefore to HR. This may also explain why other DSB repair pathways that do not use extensive resection, and are prolific in mitotically dividing cells, are blocked in wild-type meiosis (*i.e.,* canonical nonhomologous end joining or microhomology-mediated end-joining). Following resection, the replication protein A (RPA) complex binds and stabilizes the 3′-end ssDNA, and is promptly replaced by Rad51, which drives homology search (Sung 1994; New et al. 1998; Shinohara and Ogawa 1998; Hu and Crickard 2024; Tian et al. 2024). In meiosis, homology search is targeted to the homologous chromosome, which is aided, in part, by the synaptonemal complex, which keeps homologs in proximity and excludes sister chromatids from most repair (Holliday 1977; Page and Hawley 2004). Once homology is identified, strand invasion of the Rad51-ssDNA filament displaces one strand of the target sequence, generating a DNA structure known as the displacement loop. DNA synthesis and ligation convert this structure to a dHJ (Szostak et al. 1983; Schwacha and Kleckner 1995; Tian et al. 2024), while ejection of the invading strand prior to dHJ formation results in SDSA (McMahill et al. 2007). However, studies in yeast are consistent with SDSA diverging from the CO-generating pathway not only at but also prior to strand ejection (Allers and Lichten 2001). Thus, although the decision whether to eject the invading strand may be a key step in the dHJ/SDSA pathway choice, other, earlier deciding factors may be at play as well.

Multiple proteins are involved in the maturation of dHJs to COs, including the MSH-4/MSH-5 complex, a dimer that forms a ring on dHJ intermediates prior to their ligation (Kelly et al. 2000; Manhart and Alani 2016). Through the formation of this dimer, the MSH-4/MSH-5 complex stabilizes the strand invasion products and supports the formation of a mature dHJ (Snowden et al. 2004; Rakshambikai et al. 2013). In *Caenorhabditis elegans*, MSH-5 is recruited to numerous DSB sites, and other factors are required to stabilize it on the few DSBs that will form COs (Kelly et al. 2000). One of these evolutionarily conserved factors is crossover site-associated-1 (COSA-1) (Yang et al. 2024). COSA-1 is a cyclin-associated protein, and in *C. elegans*, it is shown to act with CDK-2 (Yokoo et al. 2012; Haversat et al. 2022). COSA-1 is required for the selection of a small subset of sites to form COs, likely by supporting the phosphorylation of MSH-5 (Haversat et al. 2022; Yang et al. 2024). The fact that MSH-5 sites are numerous but then are whittled down to a few COSA-1-associated locations is consistent with a model in which the invading ssDNA is ejected at a location that had the potential to form a dHJ, resulting in SDSA instead. Strand ejection in SDSA requires the action of helicases: in metazoan meiosis, RTEL-1 is the only known regulator of the SDSA pathway (Youds et al. 2010), while in yeast, Srs2 plays a similar role (Liu et al. 2017). As nuclei move from the distal to the proximal germline, they are exposed to different signaling cascades, some intrinsic and some extrinsic, which are activated in different regions of the germline (*e.g.*, Notch, IP3, MAPK, and CHK-2, respectively [Austin and Kimble 1987; MacQueen and Villeneuve 2001; Meier and Ahmed 2001; Bui and Sternberg 2002; Lee et al. 2007; Baylis and Vazquez-Manrique 2012; Tolkin and Hubbard 2021; Kimura and Motegi 2025]). Some of these signaling cascades are implicated in DSB formation and repair (Hayashi et al. 2007; Kim et al. 2015).

In *C. elegans* meiosis, both dHJ and SDSA pathways are active, with each nucleus averaging 6 COs (1 per each chromosome) and 6 to 10 times as many NCOs (Mets and Meyer 2009; Rosu et al. 2011; Saito et al. 2012; Saito and Colaiácovo 2017). COs represent the minority of DSB repair outcomes in other organisms as well (Mancera et al. 2008; Martinez-Perez and Colaiácovo 2009; Cole et al. 2012). As stated above, the standard model for meiotic DSB repair is that dHJ and SDSA diverge after strand invasion. This model implies that each DSB, regardless of when it is created, has the potential to be repaired through the dHJ or SDSA pathway, with the only exception being CO interference (Saito and Colaiácovo 2017; Zickler and Kleckner 2023). CO interference is a process in which DSBs that mature into a CO suppress proximal DSBs from forming COs, targeting them for SDSA (von Diezmann and Rog 2021). This mechanism implies that the chromosomal location of the DSB determines the dHJ/SDSA pathway choice. However, CO interference does not exclude additional layers of regulation. For example, the timing of DSB formation may influence the likelihood of being repaired by dHJ (CO) vs SDSA (NCO). Our previous finding indicated that, indeed, the CO/NCO decision is influenced by the timing of DSB formation (Hicks et al. 2022). Another study from our lab provides additional evidence supporting a model where the SDSA and dHJ pathways diverge prior to strand invasion (Oberlitner et al. 2025). These studies are consistent with a model in which the timing of DSB formation affects the mode of repair, in addition to CO interference that executes the dHJ/SDSA decision in a positional manner.

In *C. elegans,* DSBs are formed continually from meiotic entry (transition zone [TZ]) to mid-pachytene (MP) to late-pachytene (LP) transition (Hicks et al. 2022). Based on the timing of DSB formation, 2 distinct types of DSB subpopulations form: early DSBs, which form from meiotic entry to MP stage of meiosis, and late DSBs, which form from the middle of MP to LP entry. Early DSBs do not form COs and are destined to form NCOs, while late DSBs can generate both COs and NCOs. Although the distinction between early and late DSBs has not yet been examined in other systems, this finding relates to 2 yeast studies (Joshi et al. 2015; Sandhu et al. 2020). In these studies, the timing of DSB formation in yeast correlates with the selective use of repair templates: early DSBs preferentially employ sister chromatids, while late DSBs utilize homologous chromosomes. Thus, these studies are also consistent with the notion that DSBs formed at different times in meiosis are repaired differently, although in the yeast system, the effect is on the repair template and not on the pathway used.

To identify the point in meiotic prophase I in which there is a transition from early-to-late DSB formation on a developmental scale, we first characterized the changes in germline meiotic stages in our system from L4 to 3-day-old adult. We determined that an increase in the number of rows of pachytene is mainly contributed by germline growth. In the process of obtaining a more accurate depiction of nuclear movement rate in the germline, we determined that the movement rate of the nuclei in the gonad gradually slows from TZ to LP. We established that the DSBs that produce COs are generated in the second half of the pachytene region, regardless of germline age. This indicates that the region required for CO formation (late DSB region) expands through development, but the fraction of nuclei in the late DSB region from the total pachytene nuclei remains constant. Thus, the activation of the pro-CO pathway coincides with the peak of RAD-51 foci numbers in the middle of pachytene as the *C. elegans* germline grows.

## Materials and methods

### *C. elegans* strains

All *C. elegans* strains used in this study were cultured on nematode growth medium (NGM) following standard conditions (Brenner 1974) at 20 °C (except for the experiments relating to the degradation by 3-indoleacetic acid [auxin], when worms were grown on auxin-containing plates, for the periods indicated). The *E. coli*  OP50 strain was used to feed the *C. elegans* strains. Table 1 reports the genotypes of all mutant alleles and tagged lines used in this study.

**Table 1.** *C. elegans* strains.

C. elegans genotype	Lab	Strain line
*OLLAS::cosa-1 III; spo-11::AID::3xFLAG ieSi38 IV*	Silva lab	NSV420
*dendra2::H2B; OLLAS::cosa-1 III; spo-11::AID::3xFLAG ieSi38 IV*	Smolikove lab	SSM695

### Photoconversion assay

The strain used for all photoconversion experiments was *OLLAS::cosa-1 III; spo-11::AID::3xFLAG ieSi38 IV; dendra2::H2B V.* Photoconversion experiments were conducted by either Nikon SoRa spinning disk confocal (Fig. 3 and Supplementary Fig. 1c) or Leica SP8 confocal microscope (Supplementary Fig. 1) and were based on stain and protocols developed by others (Rosu and Cohen-Fix 2017). Twenty-four, 36, and 48 h post-L4 stage worms were used in the photoconversion experiments by Nikon SoRa spinning disk confocal microscope, and 12 h post-L4 stage worms were done by Leica SP8 confocal microscope. To prepare the worms for photoconversion, they were placed in 10 μL M9 buffer (42 mM Na_2_HPO_4_, 22 mM KH_2_PO_4_, 86 mM NaCl, and 1 mM MgSO_4_) on a dish plate and then held in place with Polybead (Polysciences, Inc) and covered with a 10% agar pad. Photoconversion was performed with a 60X/1.42 NA oil objective, where ∼3 rows of nuclei in the germline were selected as the region of interest (ROI). Photoconversion was performed at 3 different locations TZ, MP, and LP, as shown in Fig. 3 and Supplementary Fig. 1, with UV405 laser light for both systems. Photoconversion was done at 0.8% power, and 8 μs dwell time for Nikon SoRa spinning disk confocal, or with 50% power and 30 intervals for Leica SP8 confocal microscope. The nuclei chosen for photoconversion were selected in the same way for both systems. To photoconvert at TZ, the rows of nuclei were counted from the pre-meiotic tip (PMT) to LP, while to photoconvert at MP or LP, the rows of nuclei were counted from LP to PMT and recorded as negative numbers. The end of LP is identified as narrowing down to 2 nuclei per row. Therefore, rows 23, 24, and 25 were selected as the ROI of TZ, rows −21, −20, and −19 were selected as the ROI of MP, and rows −13, −12, and −11 were selected as the ROI of LP. The averages of row numbers were recorded as the starting point of each location. For Nikon SoRa, the unconverted Dendra2 nuclei were observed in FITC (13% power and 300 ms exposure time), and the photoconverted Dendra2 nuclei were observed in Cy3 (50% power and 800 ms exposure time), and an immediate Cy3 emission was observed in the selected ROI after photoconversion. For Nikon SoRa photoconversion experiments, worms were recovered from the dish plate using M9 and transferred to OP50-seed NGM plates for 10 or 10.5 h before being prepared for imaging. For Fig. 3, worms were prepared using the same methodology as above prior to photoconversion. Then live imaging was performed under Cy3 (13% power and 300 ms exposure time) and Cy3 (80% power and 1 s exposure time). For Leica SP8, the unconverted Dendra2 was observed in green (488 nm laser), and the photoconverted Dendra2 was in red (552 nm laser). An immediate 522 nm emission was observed in the selected ROI after photoconversion. For Supplementary Fig. 1, Leica SP8 photoconverted worms were recovered on OP50-seeded NGM plates for 10 to 12 h before dissection. The Dendra2-tagged line was dissected and imaged by a DeltaVision microscope. Slides were fixed as described in the following section “traditional fix,” but without blocking or antibodies (only 4′,6-diamidino-2-phenylindole [DAPI] staining was used). Slides were washed with 1× phosphate-buffered saline with Tween (PBST) and then mounted with Vectashield before sealing and imaging.

### Immunostaining

In this study, strains were grown on NGM plates, which served as a control, and all worms were picked at the L4 stage and continued to grow on NGM plates, and then were dissected at different time points according to the experimental instructions.

For each slide, ∼10 worms were dissected at the head by razor blade in 10 μL sperm salts solution (50 mM PIPES, 25 mM KCl, 1 mM MgSO_4_, 45 mM NaCl, and 2 mM CaCl_2_) on an 18 × 18 mm cover-slide. Positively charged slides were then used to transfer worms by flipping the coverslips over immediately after dissection. Subsequently, the positively charged slides were placed on an ice block insulated with dry ice for at least 30 min (stored overnight in −80 °C or proceeded to the next step). Razor blades were used to take off the coverslips after freezing the charged slides on a dry ice block. In the “traditional fix” method, the slides were immediately placed in cold methanol (prechilled at −20 °C) for 1 min; in the “fix-first” method, instead, the slides were placed in cold methanol for 2 min followed by 10 cycles of “dip and lift” in cold acetone. Slides were then fixed at room temperature for 30 min by adding 800 μL of 4% PFA fix in a humid chamber. 4% PFA solution was prepared from 37% stock in different solvents: traditional fix used 1 × phosphate-buffered saline (PBS), 80 mM HEPES, 0.8 mM EDTA, and 1.6 mM MgSO_4_, whereas the fix-first used only 1 × PBS. With the exception of OLLAS::COSA-1 staining, which used fix-first, all other immunofluorescence experiments employed the traditional fix method.

For immunofluorescence, regardless of the fixation method used, slides were washed 1× PBST (1× PBS with 0.1% Tween) for 10 min and blocked in 0.5% BSA dissolved in 1× PBST for 2 h. Incubation of 60 μL primary antibodies was conducted overnight at room temperature. The next day, 1 wash was performed in 1× PBST for 10 min, and then 50 μL secondary antibodies was applied for 2 h at room temperature in the dark. Both primary and secondary antibodies were diluted in 1× PBST and covered with parafilm. The slides were incubated in the humid chamber. One wash was performed in 1× PBST for 10 min before counterstaining the DNA with DAPI for 10 min at room temperature in the dark, using a 1:10,000 dilution of 5 mg/mL DAPI stock solution (Sigma) in 1× PBST. The slides were washed once in 1× PBST for 10 min to destain, mounted in Vectashield (Vector Laboratories), and then the 22 × 22 mm cover-slides were sealed with nail polish.

### Whole-worm ethanol fixation and DAPI body analysis

For experiments in Supplementary Fig. 3c and d, after worms were picked at L4 stages, and grown at 20 °C as per experimental time points and conditions, they were placed on an uncharged slide (Surgipath Leica) in a drop of M9 buffer to remove the bacteria. Then M9 was removed with filter paper, and worms were fixed with 8 μL absolute ethanol. After the worms were completely dried, 8 μL of 1:10,000 dilution of DAPI stock solution in M9 was added and mounted with Vectashield. Finally, slides were sealed with nail polish for imaging by the DeltaVision microscope.

### Auxin-induced degradation assay

For auxin-induced degradation experiments, auxin plates were prepared by using a standard NGM recipe to which 1 mM auxin dissolved in absolute ethanol was added. Notably, the auxin plates could be stored in the dark at 4 °C for up to a month, and the fresh auxin stock solution was diluted, so the final concentration was 1 mM in the NGM media. In Fig. 5 and Supplementary Fig. 3, L4 stage worms of the indicated genotype were picked onto NGM plates and cultured for the specified time as the experiment required. The worms were then transferred to auxin plates and grown in the dark for the indicated time. For DAPI body counting experiments, in Supplementary Fig. 3c and d, the worms also needed to be recovered on NGM plates after exposure to auxin. In Fig. 5 and Supplementary Fig. 3, L4 stage worms of the indicated genotypes were plated on NGM plates as negative controls and on auxin plates as positive controls.

### Image acquisition

All images of fixed samples were taken by the DeltaVision wide-field fluorescence microscope (GE Lifesciences) with z-stacks at 0.2 mm thickness. 100×/1.4 NA oil Olympus objective was used for RAD-51 foci, COSA-1 foci, and DAPI body counting in Figs. 4 and 5 and Supplementary Fig. 3. 60×/1.42 NA oil Olympus objective was used for the row of nuclei, and the rate of nuclear movement experiments in Supplementary Fig. 1. Images were deconvolved at a conservative ratio and analyzed with SoftWoRx software (Applied Precision).

### Quantification of the number of rows of nuclei in a germline, RAD-51 foci numbers, COSA-1 foci numbers, and DAPI body numbers

For row and RAD-51 foci counting experiments in Figs. 2 and 4, *spo-11::AID* strains were grown on NGM plates and dissected at ages L4, 8 h post L4, 12 h post L4, 1-day-old, 1 and a half-day-old, and 2-day-old. The number of nuclei analyzed for OLLAS::COSA-1 and RAD-51 foci, the number of germlines analyzed for row counting and nuclear movement, and the number of D-1 oocytes analyzed for DAPI body are reported in the specific figure legends.

For quantifying the number of nuclear rows in Fig. 2, SYP-1 was used to determine the row number in which meiotic entry takes place and the transition from TZ to EP and EP to MP. SYP-1 was expressed as foci in TZ and shown as chromatin structure in pachytene. The total number of rows of nuclei was counted throughout the germline until the number of nuclei in each row was reduced to 2, which is the start of diplotene.

To quantify the number of nuclear row movements following photoconversion, the row numbers of photoconverted Dendra2 (in red) were recorded after recovering for 10 to 12 h on OP50-seeded NGM plates. Depending on the different system of photoconversion under UV405 light, either forward or reverse counts were recorded as described in the photoconversion assay. The row movement was calculated based on the starting (photoconversion assay) and ending positions. Then, the rate of nuclear movement was calculated as the total row movement divided by the time from photoconversion to post-recovery analysis (recovery time).

Quantification of all foci was performed following deconvolution of the images in SoftWorx software. In short, cytoplasmic background foci were adjusted to fewer than 5 foci per image before the foci were quantified. For quantification of RAD-51 foci, data were presented both by binning into zones as Supplementary Fig. 2, as seen in prior publications (Colaiácovo et al. 2003; Hicks et al. 2022), and by nuclear rows (Fig. 4b to g). Graphing of the data by rows aided in identifying the peak of RAD-51 foci at different stages.

For quantification of OLLAS::COSA-1 foci, zone 7 was analyzed. Quantification of DAPI bodies was done in the proximal diakinesis oocyte (D-1 oocytes) of gonads from worms according to the experimental conditions in Supplementary Fig. 3c and d.

### Statistical analysis and regression

GraphPad Prism software was used for statistical analysis (2-tailed Mann–Whitney test). Comprehensive information on the statistical methods, sample sizes, and corresponding *P* values can be found in the respective figure legends. For regression plots (Fig. 5d and e), Prism was used (linear regression straight line), and slopes were obtained from best-fit values with the Extra sum-of-squares F test. The data points for the regression analysis were calculated based on the experimental data obtained in this manuscript. For each time point (*t* = time point): ((total rows at *t*) − (rows of movement at *t*))/total rows at *t* = % of germline moved. The total row # was the average of the counts of total row numbers of 5 to 6 germlines at each time point. Row movement was the time of exposure multiplied by the time of movement (from Fig. 3). The “expected” values for each of the 3 models were derived from the initial value of the first time point and then stays constant always at the same # of rows from start (% reduced over time), stays constant always at the same %, or stays constant always at the same # of rows from end (% increased over time).

### Reagents

For antibodies, chemicals and software used, see Table 2. For primers used, see Table 3.

**Table 2.** Reagents/resource.

Reagent or Resource		Source	Identifier
Antibodies	Polyclonal rabbit anti-OLLAS (1:1,000)	Genscript	Cat. # A01658
	Polyclonal rabbit anti-RAD-51 (1:30,000)	Genscript	Smolikove lab
	Polyclonal goat anti-SYP-1 (1:500)		Smolikove lab
	Donkey anti-goat AlexaFluor 555 (1:500)	Invitrogen	Cat. # A21432
	Donkey anti-rabbit Alexa Fluor 488 (1:500)	Thermofisher	Cat. # A21206
Bacteria	*E. coli* OP50	CGC	OP50
Chemicals	5 mg/mL DAPI	Sigma	D9564
	Auxin	Sigma	Cat. # I3750
	Polybead	Polysciences, Inc	Cat. # 00876-15
	VECTASHIELD Antifade Mounting Medium	Vector Laboratories	Cat. # H-1000
Software	Photoshop	Adobe	
	Softworx	AppliedPrecision	
	Office	Microscoft	
	ImageJ	Fiji	
	Prism	GraphPad	

**Table 3.** PCR conditions.

Genotype	Primers	Tm	t	Expected WT	Expected mutant
*TIR1 (chr IV, ieSi38)*	GGGCAACGGGTGACGAG CGAGGGATTCTCCACCGAC	68C	20s	0 bp	260 bp
*cxTi10882, chr IV*	GATCTTGGATCCACATCTTTGGTCC CCCTGATTCTGTCAAGCCTATGAAG	64C	35s	572 bp	0 bp
*spo-11::degron*	CGGTTCCTTTCCCCTTTTG GAAATTCCGATTCAACCAGAG	60C	30s	250 bp	450 bp
*ollas::cosa-1*	AACCTGATTCGCTGCTGATA ACTTTGTATTGGTCTCGCAC	57C	20s	300 bp	350 bp
*Dendra2::H2B*	ACTTGGACTTACTAACTAACGGA GCAACTGCGTGTTCGTATA	53C	20s	0 bp	220 bp

## Results

Building on our previous findings (Hicks et al. 2022), we sought to identify the point at which the dHJ pathway is activated. In our previous work, we depleted SPO-11 using the auxin-mediated degradation (AID) system in the young adult developmental stage (Hicks et al. 2022) in the *spo-11::AID* strain (Zhang et al. 2015). In this system, the OsTIR1 F-box protein is ectopically expressed and binds an AID-tagged protein upon auxin exposure, triggering the ubiquitination of the target protein and subsequent proteasomal degradation. In our earlier work, we provided evidence that DSBs induced early in meiosis, from the TZ up to MP, are repaired as NCOs (Hicks et al. 2022). In contrast, late DSBs, induced from MP to LP entry, may be repaired as either COs or NCOs (Hicks et al. 2022).

**Fig. 1.**
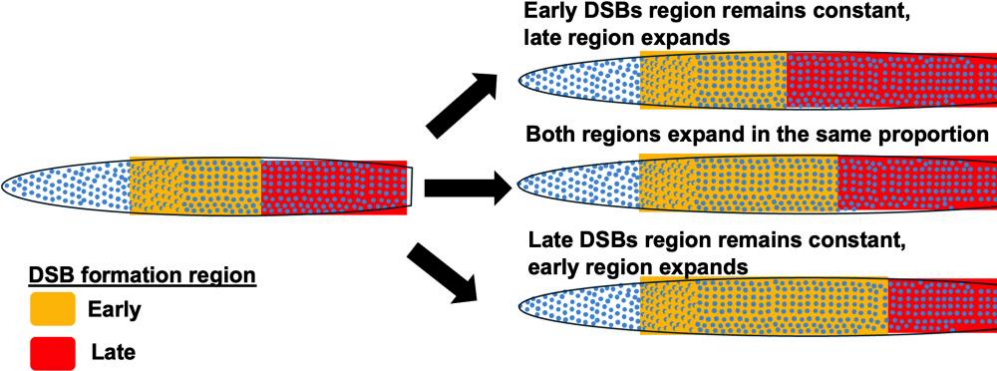
Model tested. This diagram illustrates 3 different models of how DSB regions are defined, leading to the expansion of different populations of nuclei as the germline grows. Yellow and red regions represent the early DSB region and the late DSB region, respectively. (1) Late DSB region expands (top right), (2) both regions expand in the same proportion (middle right), and (3) early DSB region expands (bottom right). Initial conditions (*t* = 0) are just prior to the addition of auxin, and the end of the experiment (*t* = analysis, when worms are taken off auxin and dissected for imaging).

One limitation of our previous work was that it relied on analysis at a single time point (Fig. 1). When L4 worms were exposed to auxin for 18 h, most of the COSA-1 foci were removed (Hicks et al. 2022). These data indicated that for L4 worms, the transition between early and late DSBs occurred in MP. As a result, it remained unclear whether the transition in repair pathway usage is triggered immediately upon reaching MP, if it depends on the total time a nucleus has spent in the germline (*e.g.,* the distance from the distal tip cell), or if it is determined by the time from an end point (*e.g.,* the distance from the proximal oocyte). Different signaling cascades can account for each one of the 3 mechanisms (see introduction). All 3 options were valid based on our prior analysis. Discriminating between the models has implications for the origin of the signal for the transition from early-to-late DSBs and, therefore, its future identification (outside of the scope of this manuscript).

The *C. elegans* germline undergoes continuous meiosis throughout much of the organism's adult life, during which the germline grows and actively produces oocytes (Tolkin and Hubbard 2021; Davis et al. 2023). We can make predictions for each of the 3 models proposed for the signal for early-to-late DSB transition (Fig. 1). If the signal is distal (from the mitotic region) it will lead to maintenance of the early DSB region at a constant size and therefore expansion of the late DSB region during development (top of Fig. 1), or if the signal is proximal (from the oocytes) it will lead to maintenance of the late DSB region at a constant size and therefore expansion of the early DSB region during development (bottom of Fig. 1). However, if the signal for the early-to-ate DSB transition always occurs in MP then both regions will expand equally (middle of Fig. 1). This model can be tested by applying the same methodology as we published (Hicks et al. 2022) but in different developmental stages (correlating with different initial germline sizes).

To this end, we first needed to define several parameters at 6 different time points: (i) characterize the growth dynamics of the germline in adult worms to determine how the number of nuclei and the distribution of meiotic stages change during the first 2 days of adulthood, (ii) the distribution of recombination intermediates (as proxy for DSBs), and (iii) the rate of progression of nuclei in the germline. No comprehensive study of all our time points is available for the first and second parameters. Moreover, although the third parameter was previously defined (mitotic: Rosu and Cohen-Fix 2017, early-to-mid prophase: Crittenden et al. 2006; Jaramillo-Lambert et al. 2007; Morgan et al. 2010), the methodology used for defining the rate of nuclear movement in meiosis is only accurate for early meiotic stages, while our experimental setup requires more precision in later stages (MP-LP). Furthermore, these parameters were never defined throughout development in the *OLLAS::cosa-1; spo-11::AID::3xFLAG* (hereafter: *spo-11::AID)* strain used for this approach. The analysis below was performed in this genetic background, in which, without auxin exposure, the germline is indistinguishable from wild type, and, with auxin exposure, shows a *spo-11* phenotype (lack of COs). Importantly, while SPO-11 is required for DSB formation, the lack of DSBs in the null mutant or the depleted germline (AID) does not affect meiotic progression or germline size in *C. elegans* (Dernburg et al. 1998; Zhang et al. 2015; Hicks et al. 2022). Our previous work also suggested that SPO-11 degradation is rapid, and the time required for RAD-51 disappearance is mainly reflective of the duration of homology search and RAD-51 disassembly, which was also supported by the kinetic analysis of RAD-51 disappearance performed by others (Hicks et al. 2022; Hamrick et al. 2026). The 6 time points were selected within the 3 fertile days of *C. elegans*, when sperm are not yet depleted, and stem cells proliferate (Tolkin and Hubbard 2021). At these time points, germline growth is significant, allowing us to test our hypothesis, yet worms are physiologically similar and do not show significant hallmarks of aging (Raices et al. 2021; Tolkin and Hubbard 2021).

### Germline growth is mainly contributed to by an increase in the number of nuclear rows of pachytene

In *C. elegans*, most germline nuclei are interconnected by a shared cytoplasm in a tube-like syncytium (rachis) and are arranged at the periphery of the tube (Hubbard 2007). The nuclei form rings and move from the distal to the proximal end as the germline proliferates. From a side view, this ring appears as rows of nuclei (∼8 on each side), and germline size is frequently quantified using the number of nuclear rows, which increases during development. The terminology of nuclear rows is the standard unit for describing the rate of nuclear movement in the germline and was established by others (for example, Crittenden et al. 2006; Morgan et al. 2010; Hubbard and Schedl 2019).

The *C. elegans* germline can be divided into several stages from the distal to proximal end. The germline distal region contains PMT nuclei, and it is where proliferating progenitor cells continuously mitotically divide to ensure a continuous population of cells entering meiosis (Hubbard and Greenstein 2005). As nuclei move proximally, they enter the TZ, where meiotic prophase I is initiated and endogenous DSBs are generated by SPO-11 and repaired via SDSA. At TZ, chromosomes form a unique crescent-like morphology, as homologous chromosomes begin to pair and cluster to one side of the nuclear periphery (Lui and Colaiácovo 2013). As nuclei progress through the cell cycle, they move further proximally and enter pachytene, which separates into early (EP), MP, and LP, respectively. In the pachytene stage, the synaptonemal complex is fully assembled (MacQueen et al. 2002), and meiotic DSBs continue to form. These DSBs may also utilize the dHJ pathway, generating 6 COs per nucleus by the end of this stage (Saito and Colaiácovo 2017; Gartner and Engebrecht 2022). In the following stage, diplotene, the germline bends, and the number of nuclei per row shrinks to 2, and chromosomes are desynapsed. Finally, at diakinesis, 6 homologous chromosome pairs (bivalents) are visible, held together by chiasmata formed by the 6 COs, stabilized by sister chromatid cohesion (Saito and Colaiácovo 2017). For the purposes of this study, we have focused on TZ, EP, MP, LP, and diakinesis stages of meiotic prophase I.

Our methodology involves exposing *spo-11::AID* worms to auxin for a distinct number of hours. Based on the rate of nuclear progression, we can extrapolate where meiotic DSBs were removed. To perform this extrapolation, we need to identify the rate of nuclear movement in meiotic prophase I. As the rate of nuclear movement is defined using the unit of rows/hour, it is critical to identify the number of nuclear rows in each stage analyzed in the *spo-11::AID* strain. There has been no quantitative analysis of germline growth in the *spo-11::AID* strain, and a limited description in other wild-type strains. These data are particularly absent regarding changes in the number of rows of nuclei and differential expansion of the meiotic stages within the fertile days of the worms. For example, germline growth was evaluated and computationally simulated in wild type, without analysis of meiotic stages (Atwell et al. 2015), or analyzed during aging, but not within the fertile days of the worms (Kocsisova et al. 2019). Thus, we do not know what the changes in the size of meiotic stages in the gonads are, and if these changes are restricted to particular meiotic stages as the worms grow.

**Fig. 2.**
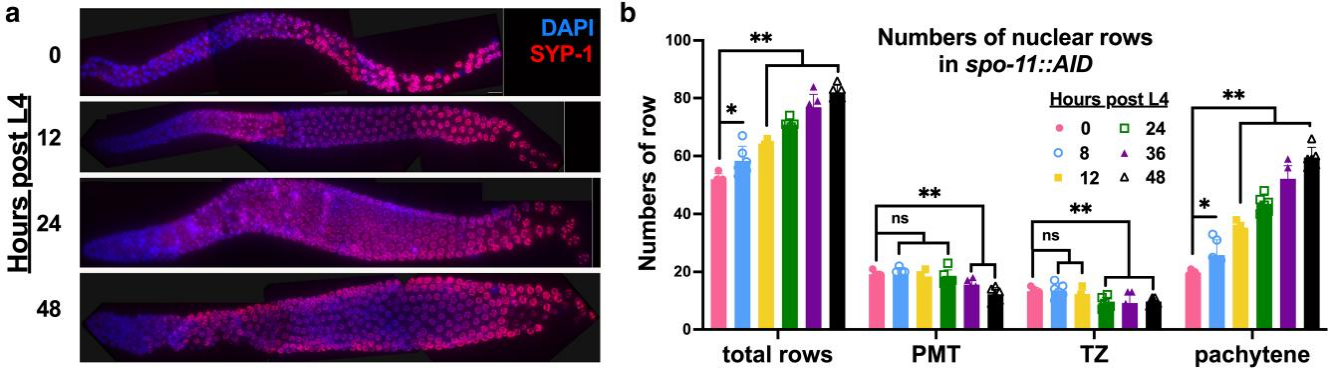
Germline growth is mainly contributed to by an increase in the row numbers of pachytene. a) Representative images of gonads from 4 different time points during L4 to early adult development of *spo-11::AID; ollas::cosa-1* worms at the indicated times. SYP-1, a marker for the synaptonemal complex, was used to determine meiotic entry and help differentiate TZ from EP. Blue—DAPI, red—SYP-1. Scale bar applies to all images in the panel, 10 μm. Asterisks indicate statistical significance calculated by the Mann–Whitney test (^∗∗^*P* < 0.01, and ^∗^*P* < 0.05; ns—not significant). b) Quantification of the number of nuclear rows in *spo-11::AID; ollas::cosa-1* worms at different time points during development. All analyses were performed in at least 5 gonads. Data are shown as mean ± SEM.

We have sought to identify the number of nuclear rows in each meiotic stage and the PMT at 6 time points in *spo-11::AID* germlines. To accurately identify TZ, we employed SYP-1 immunostaining in combination with DAPI (MacQueen et al. 2002) to visualize chromosome morphology and identify meiotic stages throughout the germline (Fig. 2a). SYP-1 is a major component of the synaptonemal complex—a proteinaceous structure that forms between homologous chromosomes during meiotic prophase I (MacQueen et al. 2002). The level of assembly of the synaptonemal complex and chromosome organization (DAPI) is used to determine the stage at which a nucleus resides in meiotic prophase I. As shown in Fig. 2a, SYP-1 chromosomal association is absent in PMT, indicating that synapsis has not yet initiated in this distal region. In the TZ, SYP-1 begins to appear as discrete puncta, corresponding to initial synapsis events. In the pachytene region, SYP-1 staining becomes continuous along the length of chromosomes, reflecting full synapsis (MacQueen et al. 2002). Using these markers, we were able to distinguish meiotic stages reliably and quantify the number of nuclear rows in 6 time points from L4 to 2-day-old adult (Fig. 2a).

For this, *spo-11::AID* worms were cultured on standard NGM media and dissected at multiple time points post-L4 (see *Materials and methods*). Our results reveal that, as expected, the total number of nuclear rows in the germline increases significantly over time. At the L4 stage, worms have approximately 50 nuclear rows, which increases to around 70 rows by the time they become 1-day-old adults and further expands to over 80 rows in 2-day-old adults (Fig. 2b). Interestingly, this increase is not uniformly distributed across all germline regions. The PMT and TZ show relatively stable row counts, with only minimal fluctuations observed between 1.5-day-old and 2-day-old adults. In contrast, the most dramatic growth, which accounts for the overall growth in germline size, is observed in the pachytene region. Initially, the pachytene region comprises about 20 rows at the L4 stage. This increases to over 40 rows by day one and exceeds 60 rows by day 2 of adulthood (Fig. 2b).

These results suggest that the overall expansion of the germline is primarily driven by the growth of the pachytene region, which more than triples in size over 48 h. These findings provide a quantitative foundation for interpreting stage-specific recombination dynamics and pave the way for future studies exploring how the duration of time spent in pachytene affects meiotic DSB repair.

### The movement of germline nuclei gradually slows as they progress through pachytene

To accurately define the time windows corresponding to different meiotic stages in SPO-11 depletion experiments, a precise analysis of nuclear migration dynamics is required. The dynamics of nuclear movement in the germline have been tracked using nucleotide analogs (Cy3/Cy5-dUTP, BrdU, or EdU labeling), revealing a consistent progression of ∼1 cell row per hour across conditions, and unaffected by genetic background, age, or auxin treatment (Crittenden et al. 2006; Jaramillo-Lambert et al. 2007; Morgan et al. 2010; Hillers et al. 2017; Hicks et al. 2022). These methods, by definition, provide only average movement, always from meiotic entry (pulse time point) to another germline region (chase time point). If the movement rate of nuclei varies throughout a region of meiotic prophase I, these methods would not be able to identify it. For our analysis, the movement rate at mid-to-late pachytene (“late DSBs” region) is the most relevant, but this stage is the furthest away from the mitotic zone, and what is typically and reliably analyzed for nuclear movement using the methods indicated above.

A more precise approach was introduced in recent years to label and track germline nuclei: photoconversion of the fluorescent protein Dendra2. This technique allows for real-time, longitudinal observation of nuclear movement within individual worms. Dendra is a photoconvertible fluorescent protein that undergoes a green-to-red shift upon exposure to UV-violet or intense blue light (350 to 420 nm or 488 nm; Gurskaya et al. 2006). This conversion is irreversible, allowing for the precise marking of nuclei at a given moment in time. After conversion, red fluorescence can be monitored to track the movement of labeled nuclei as they progress through the gonad. Dendra2 contains a single amino acid substitution (A224 V), resulting in a brighter fluorescence and more efficient maturation compared to Dendra (Chudakov et al. 2007). When fused with histone H2B, Dendra2 localizes to the nucleus, enabling clear visualization of chromatin dynamics. However, the use of Dendra2::H2B comes with one caveat: histone H2B is continuously turned over, leading to an ongoing increase in green fluorescence intensity (Rosu and Cohen-Fix 2017). As a result, the photoconverted red signal has a limited observation window before it becomes diluted by newly synthesized green-tagged H2B. Previous work has shown that the red signal remains detectable for up to 8 h following photoconversion (Rosu and Cohen-Fix 2017), although this time frame may vary depending on expression levels and imaging conditions. Their work proposed a stochastic model for *C. elegans* PMT proliferation, in which cells in the distal 6 to 8 rows of the PMT proliferate at ∼6 h/division symmetrically, while proximal proliferation gradually slows down and stops, and their daughter cells migrate out of the PMT over time due to spatial competition.

**Fig. 3.**
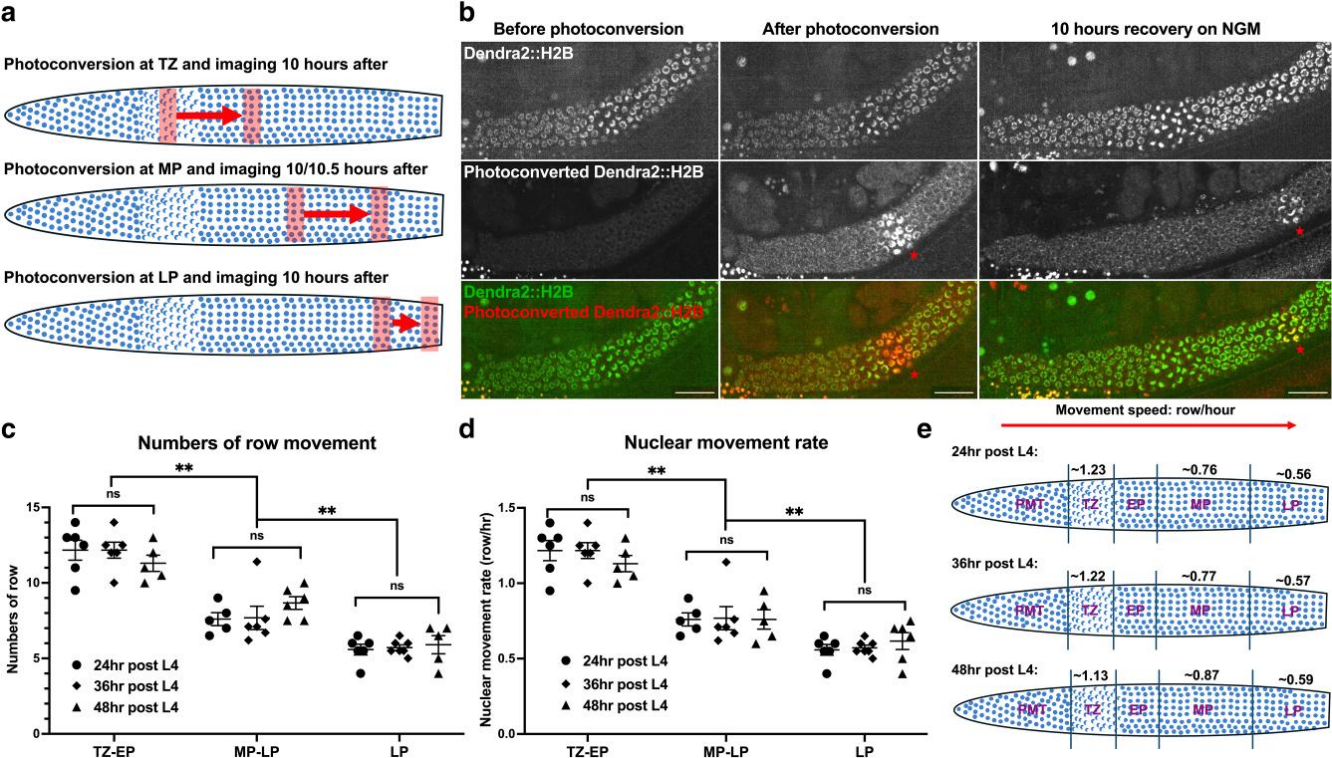
Nuclear movement gradually slows down through the germline. a) Schematic representation of the experimental setup of photoconversion. Three experiments were conducted (with different starting ages of worms), investigating 3 different germline regions. Red boxes (on the left) represent the photoconverted region and the imaged region (on the right). b) Representative images of gonads at TZ before photoconversion, after photoconversion, and 10 h of recovery on NGM after photoconversion. Green—Dendra2::H2B, red—Photoconverted Dendra2::H2B. Scale bar applies to all images in the panel, 20 μm. c) Quantification of nuclear movement by rows moved from photoconverted time to analysis time. d) Quantification of nuclear movement rate in the 3 time points of the 3 regions selected (as in a). e) Schematic representation of the results obtained from d. All analyses were performed in at least 5 gonads and in biological replicates. Data are shown as mean ± SEM. Asterisks indicate statistical significance calculated by the Mann–Whitney test (ns—not significant, ∗∗*P* < 0.01).

In our current study, we adopted the application of Dendra2 photoconversion technology for spatiotemporal resolution in the meiotic regions of the germline (Fig. 3a and Supplementary Fig. 1a). We selected 3 ROIs, each consisting of 3 nuclear rows and located in different stages of meiosis (Fig. 3a and *Materials and methods*). This targeting strategy allowed for more accurate measurement of nuclear movement rates during different stages of meiotic prophase I, removing variability caused by different entry times into meiosis. Remarkably, we observed that the red fluorescence signals were still detectable 10 to 12 h after photoconversion (Fig. 3b and Supplementary Fig. 1b). This extended window allows us to monitor the progression of nuclei in the germline more precisely.

Through this approach, we were able to quantify changes in the rate of nuclear movement as a function of developmental stage (by dividing total row movement by the time from photoconversion to post-recovery imaging). Photoconversion analysis in Dendra2::H2B expressing worms revealed a gradual deceleration of nuclear movement through the germline over time (Fig. 3c and d and Supplementary Fig. 1c). There was no statistical difference between data obtained for worms 12, 24, 36, or 48 h post-L4 (Fig. 3c and d and Supplementary Fig. 1c), or with different microscopy systems (Supplementary Fig. 1c). This indicates that the nuclear movement rates in the first 2 days of adulthood does not change. When averaged, nuclei moved at a rate of 1.21 cell rows per hour when transitioning from TZ to EP. This rate decreased to 0.79 rows per hour as the nuclei moved from MP to LP and further slowed to 0.57 rows per hour within the LP region itself (Fig. 3e and Supplementary Fig. 1d).

In addition to tracking nuclear movement, we measured the width of each cell row within the gonad and found that the average diameter of the rows (total length of the region divided by row numbers) significantly increased from TZ-EP (4.28 μm) to LP (5.86 μm) (Supplementary Fig. 1e). This is consistent with the visual observation of the increase in nuclear size as nuclei move proximally. Therefore, it is possible that nuclear movement slows down in rows/hour measurement units due to the increase in nuclear size (increased diameter) as they progress in meiosis. To account for that, we also examined the distance traveled per hour. When correlating the rate of movement with distance measurements, we still observed a decrease in nuclear movement in LP (∼40 μm/h) compared to earlier stages (∼56 μm/h) (Supplementary Fig. 1f).

In summary, our findings reveal stage-specific variation in nuclear movement during meiotic prophase I in *C. elegans* using an optimized photoconversion method. These observations, benefiting from the long-term tracking of individual nuclei, have opened new avenues for investigating regulatory mechanisms governing meiosis throughout the lifespan of the organism.

### The peak of RAD-51 foci numbers per nucleus is consistently found in the middle of the pachytene stage of meiotic prophase I, regardless of the length of the germline

To investigate the dynamics of DSB formation and repair during meiosis in *C. elegans*, we analyzed the spatial and temporal distribution of RAD-51 foci through the germline. Since direct visualization of SPO-11 is currently not feasible in worms (as well as in many other organisms), RAD-51 localization serves as a reliable proxy for DSB formation. Previous studies have shown consistent numbers and distribution of RAD-51 foci numbers between the gonads of wild-type genotypes, with minor quantitative differences due to staining conditions and/or antibody sources (*e.g.,* MacQueen et al. 2005; Guo et al. 2022; Láscarez-Lagunas et al. 2022).

**Fig. 4.**
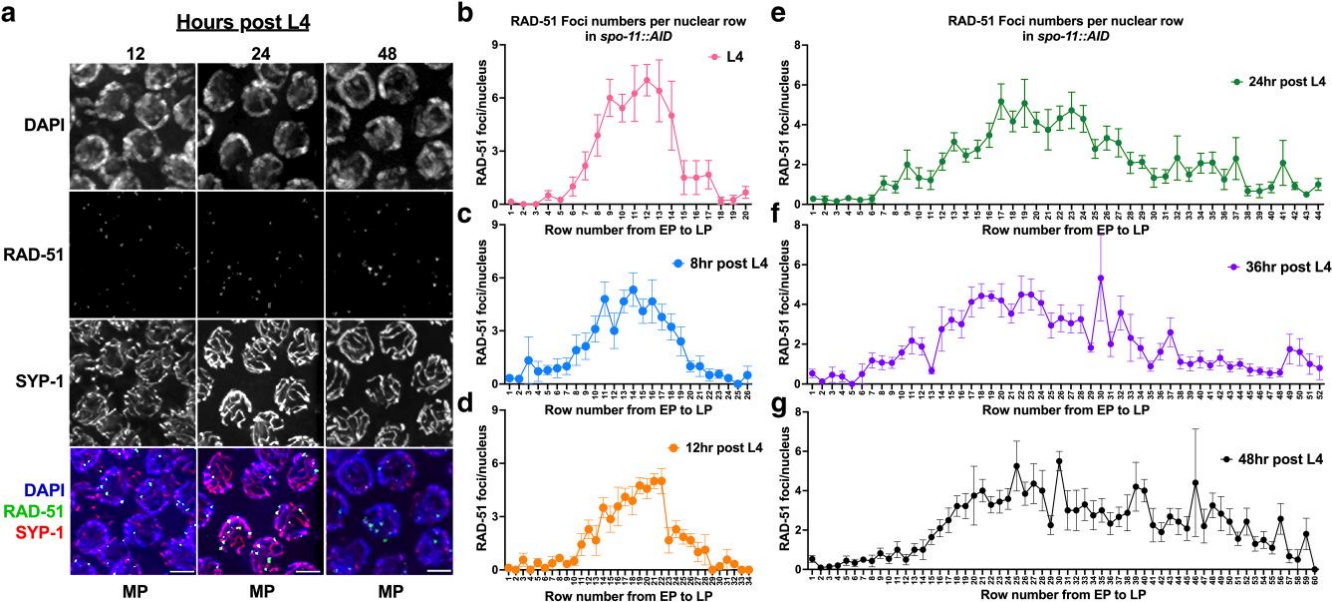
RAD-51 foci numbers peak in mid-pachytene, regardless of the time points during development of *spo-11::AID* worms. a) Representative images of nuclei from differently staged worms at the indicated times, stained for RAD-51/SYP-1 (blue for DAPI, green for RAD-51, and red for SYP-1). Scale bar applies to all images in the panel, 3 μm. b) to g) Quantification of RAD-51 foci numbers per nucleus per nuclear row from EP to LP in *spo-11::AID; ollas::cosa-1* worms at the indicated time points (b: L4, c: 8 h post L4, d: 12 h post L4, e: 24 h post L4, f: 36 h post L4, and g: 48 h post L4). EP: early pachytene; LP: late pachytene. All analyses were performed in at least 3 gonads. Data are shown as mean ± SEM.

In our experiments, we quantified RAD-51 foci across distinct zones of the germline (see *Materials and methods*) in worms at various adult stages. Immunofluorescence for RAD-51 was performed at 6 time points post-L4 (Fig. 4 and Supplementary Fig. 2). At all time points, SYP-1 staining shows well-defined, elongated tracks consistent with fully synapsed chromosomes, suggesting successful homolog pairing and synaptonemal complex assembly (Fig. 4a, and as by MacQueen et al. 2002). DAPI staining in blue revealed the chromosomal morphology, and RAD-51 foci were observed from TZ to LP, appearing as discrete puncta. In 24 h post-L4, we observed peak RAD-51 foci/nucleus in zone 5 with approximately 4 foci/nucleus on average (Supplementary Fig. 2b), corresponding to MP, consistent with previous observations (Hicks et al. 2022). This confirms that RAD-51 foci reach maximal abundance during MP under normal developmental conditions. To improve resolution, we also analyzed the number of RAD-51 foci/nucleus on a per row basis across the pachytene region. This fine-scale quantification revealed that, regardless of germline length or developmental stage, the peak RAD-51 foci consistently occurred near the center of the pachytene zone, corresponding to the middle of MP (Fig. 4b to g and Supplementary Table 1). At earlier time points (0, 8, 12, and 24 h post-L4), the distribution of RAD-51 foci/nucleus sharply peaked, as shown in Fig. 4b to e and Supplementary Table 1. In contrast, for the older worms (36 and 48 h post-L4), the distribution appeared broader, and the peak became less obvious, as shown in Fig. 4f and g and Supplementary Table 1. These findings suggest the spatial localization of RAD-51 foci is maintained as the germline develops and the number of rows increases. In older worms (36 and 48 h post-L4, Fig. 4f and g), RAD-51 levels were slightly reduced (Supplementary Table 1), as previously observed (Raices et al. 2021).

### Pro-CO DSB formation compatible region extends during development but reproducibly corresponds to the proximal half of the pachytene region

**Fig. 5.**
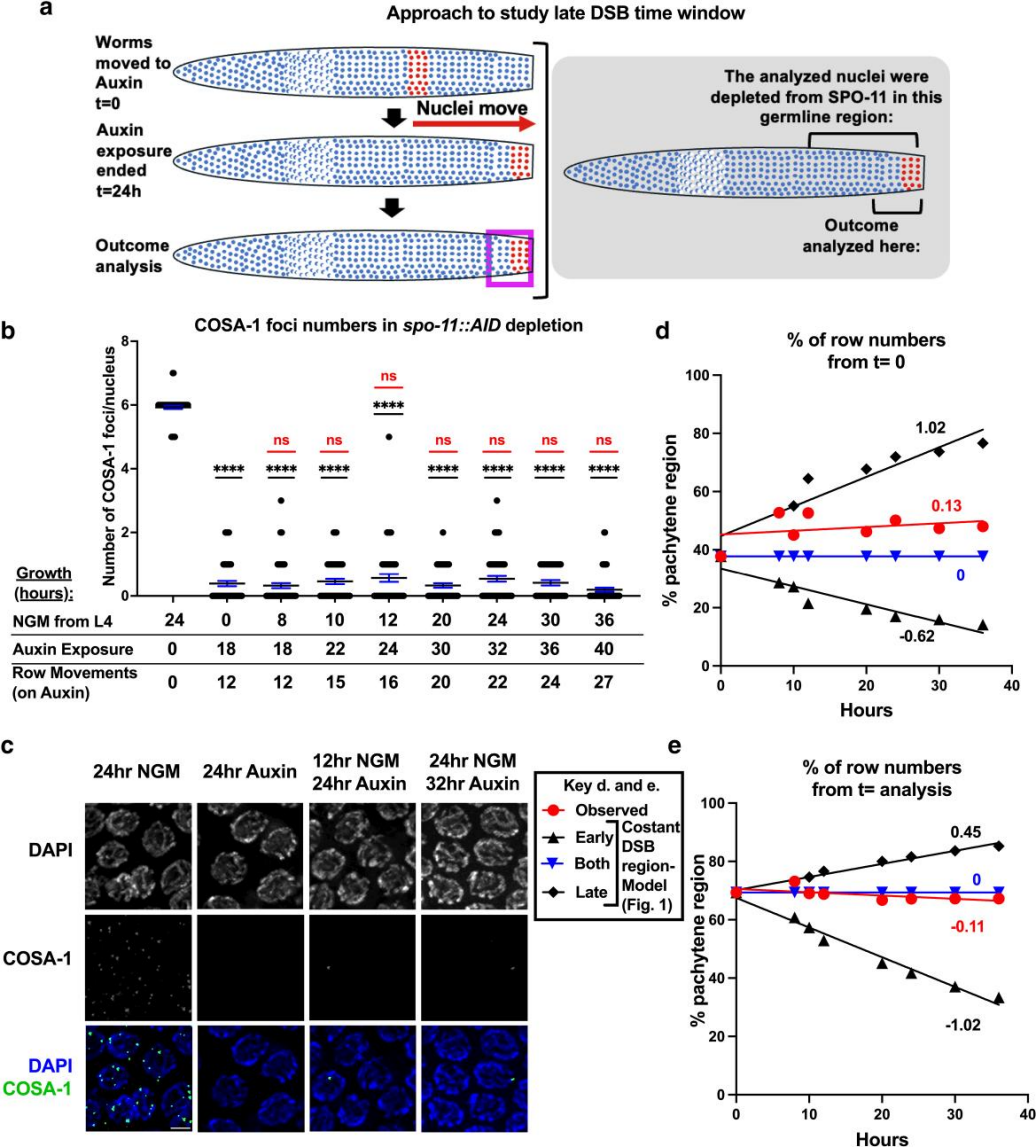
Late DSB region expands during development of spo-11::AID worms. a) Cartoon representing the system used in the study of the late DSB region. An example of experimental design with L4 worms grown on auxin plates for 24 h. b) Quantification of COSA-1 foci per nucleus in LP at 9 time points during development of *spo-11::AID; ollas::cosa-1* worms. LP: late pachytene. All analyses were performed in biological replicates. Data are shown as mean ± SEM. Asterisks indicate statistical significance calculated by the Mann–Whitney test (****P < 0.0001, ns—not significant; black represents comparisons with L4 grown on NGM plates for 24 h; red represents comparisons with L4 grown on auxin plates for 18 h. c) Representative images of nuclei from 3 time points during development of *spo-11::AID* worms, stained for COSA-1 (blue for DAPI, green for COSA-1). Scale bar applies to all images in the panel, 3 μm. d) and e) Linear regression analysis of observed data and 3 possible parameter sets based on the models in Fig. 1. From the beginning to the end of each exposure time, the germline grows, and row numbers change. We therefore need to consider the relative position of each nucleus based on the initial conditions (*t* = 0) or the end of the experiment (*t* = analysis). For % row calculations, row numbers when *t* = 0 (d) or *t* = analysis (e) were used. Red circles represent experimental (observed) data, black triangles represent early DSB region constant—expected data, blue reverse triangles represent middle DSB region constant—expected data, and black rhombus represents late DSB region constant—expected data. The numbers next to the slopes indicate the slope. For *t* = 0, the 95% CI values are: observed: −0.2488 to 0.5051, expected early −0.8432 to −0.3880, and expected late 0.6398 to 1.390. For *t* = analysis, the 95% CI values are: observed: −0.2420 to 0.01617, expected early −1.167 to −0.8675, and expected late 0.3834 to 0.5159. Expected both doesn't have CI values because it is a perfect line with 0 slop.

Using acute and reversible depletion of SPO-11 via the AID system (Zhang et al. 2015), we demonstrated that SPO-11-induced DSBs from TZ through LP, and that the timing of DSB formation during *C. elegans* meiosis influences the repair outcome (Hicks et al. 2022). DSBs generated during early meiotic stages (TZ to MP) are “early DSBs,” which are generally resolved as NCOs. In contrast, DSBs formed from MP to LP are “late DSBs,” which can generate both COs and NCOs (Hicks et al. 2022, 2024). However, we did not know what dictates the transition between the 2 repair modes. The experimental framework applied here is similar to what we have executed previously (Hicks et al. 2022, 2024). For example (Fig. 5b), if the *spo-11::AID* germline is exposed to auxin for only 18 h, then analyzed in late pachytene (via COSA-1), that means these nuclei were depleted from SPO-11 for the last 12 nuclear rows (based on Fig. 3d: 0.68 [0.79 to 0.57 rows/hour]). This time window examines DSBs that form at or right after the peak in RAD-51 foci numbers per nucleus.

In wild-type worms, each COSA-1 focus marks a CO, with 6 COs typically observed (Yokoo et al. 2012). In our experiments, multiple auxin exposure time windows were applied for *spo-11::AID* worms at different time points from the start of exposure (Supplementary Table 2) to identify the minimal time window that will result in the removal of most of COSA-1 foci for each age group at the time of analysis (Fig. 5a to c). As shown in Fig. 5b, quantification of COSA-1 foci/nucleus revealed 6 foci/nucleus under control conditions (24 h NGM), consistent with wild-type levels of CO formation (Yokoo et al. 2012; Saito and Colaiácovo 2017). Upon exposure to auxin for 18 or 24 h (ns from each other), COSA-1 foci/nucleus were nearly absent (Fig. 5b, Supplementary Fig. 3a, and Supplementary Table 2), confirming that removing SPO-11 only from the last ∼half of germline pachytene nuclei is sufficient suppression of pro-CO DSB, as we previously observed (Hicks et al. 2022). To test our model (Fig. 1), we also examined worms at various time points in which the pachytene region grows during development (Fig. 2). By examining different time windows of auxin exposure, we identified that the minimum time window for CO removal increases as the germline grows (Fig. 5b and Supplementary Table 2). To validate that loss of COSA-1 is indicative of loss of COs, we also examined DAPI body numbers in diakinesis. Germlines not exposed to auxin, as wild-type germlines produce 6 DAPI bodies per oocyte (6 bivalents), while loss of COs leads to the formation of 12 DAPI bodies at diakinesis (12 univalents) (Dernburg et al. 1998). When focusing on the 12-h post-L4 time point, we also observed that nuclei that were depleted of SPO-11 in MP to LP but were not depleted when passing through diakinesis (12 to 36 h), produced oocytes with largely no COs (Supplementary Fig. 3b and c and Supplementary Table 3), as expected from the absence of COSA-1 foci (Fig. 5b).

At the conclusion of these experiments, we obtained the data that will assist in determining which of the 3 models in Fig. 1 is more likely to be accurate. We selected the 8 time points identified in Fig. 5b (0, 8, 10, 12, 20, 24, 30, and 36 h post-L4s) and calculated the point of transition from the early DSBs region to the late DSB region in terms of rows from late pachytene to diplotene transition. To obtain the minimal number of nuclear rows from the proximal region of LP that is required for CO formation we divided the minimal number of hours of auxin exposure required for COSA-1 loss (Fig. 5b) by the rate of nuclear movement in the relevant region (average of 0.68, Fig. 3e and Supplementary Fig. 1d). The number of pachytene rows in each developmental stage (Fig. 2) was used to calculate the position of the nucleus at the first hour of auxin exposure in units of % of pachytene region. As the germline grows in size while exposed to auxin, we performed the calculation for both the initial time point of auxin exposure (*t* = 0) and for the end point of exposure, when worms were analyzed (*t* = analysis). Both calculations showed linear regression with slopes closer to those expected from “both regions expand in the same proportion” and between the 2 other models (Fig. 1, Fig. 5d and e, CI = 95%, values in figure legend). While both methods of analysis were consistent with the early-to-late transition occurring in MP, the same position where we also observe a peak in RAD-51 foci numbers (50% into pachytene, Fig. 4), the 2 methods varied in the exact placement of the transition. With *t* = 0 analysis, the average position of the transition from early-to-late DSBs was at 47% from pachytene entry (±5) before, while at *t* = analysis, that value was 69% (±2). Since the peak of RAD-51 symmetrically distributes the population of RAD-51 foci, this suggests that half, or more than half, of meiotic DSBs are residing in the early DSBs region, and therefore incompetent in forming COs.

To conclude, DSBs that produce COs are generated in the second half of the pachytene region, regardless of the size of the germline. Thus, the region required for CO formation expands through development, but the fraction of this region from the total pachytene region remains constant. Our data is consistent with activation of the pro-CO pathway in MP, coinciding with the peak of RAD-51 foci numbers.

## Discussion

Our previous finding shows that the CO/NCO decision is made very early, at the time of DSB formation, and that early DSBs are destined to form NCOs, while DSBs that form later in meiosis form COs or NCOs (Hicks et al. 2022). In our current study, we determined that the transition point remains constant, around MP, regardless of the developmental time point/germline size. Through our study, we also performed a comprehensive analysis of germline growth and showed that germline expansion from L4 to 2-day-old adult is mainly contributed to by the expansion of the pachytene stage. In addition, we have discovered that the rate of nuclear movement in the germline is not uniform and that it slows as nuclei move through the germline.

### Reduction in nuclear movement during meiotic prophase I

Meiotic prophase I can be subdivided into specific stages defined by chromosome morphology and the status of the synaptonemal complex (Zickler 2006; Lui and Colaiácovo 2013): upon meiotic prophase I entry leptotene/zygotene (in *C. elegans*, which is TZ), the synaptonemal complex initiates assembly but is not fully assembled, whereas in pachytene, homologous chromosomes are aligned and tightly associated by the synaptonemal complex, through the process of synapsis. We found that nuclear movement is slower in pachytene than in TZ to EP. This may be correlated with synaptonemal complex assembly. However, synaptonemal complex formation is not fully correlated to movement rate, as movement slows from MP to LP when the synaptonemal complex is fully formed (Figs. 2 and 3). More rapid nuclear movement in EP may be due to signaling checkpoints that are active at this point and turned off as meiosis progresses (Zhang et al. 2023). Alternatively, the slowing of movement in LP may be promoted by other factors such as apoptosis, which is activated in this stage (Schumacher et al. 2001).

Unlike budding yeast, which completes meiosis within hours, meiotic prophase I in metazoans is notably prolonged and can last for days (Jaramillo-Lambert et al. 2007; Wang and Pepling 2021). We observed that the region of meiotic prophase I continuously expanded from the L4 stage through the 2-day-old adult, allowing a longer time window for DSB formation and their regulation during worm development. This expansion may correlate with the age-related impairment in DSB repair observed in *C. elegans* (Raices et al. 2021), which may be compensated by an extended pachytene stage.

### Photoconversion is a more accurate tool for studying the nuclear movement rate in living cells

Dendra2 is linked with H2B histone; therefore, we were able to observe the recruitment of newly synthesized H2B histones as green fluorescence. Our results demonstrate that red fluorescence signals remained detectable 10 to 12 h after photoconversion, exceeding the previously reported duration of 8 h (Rosu and Cohen-Fix 2017). This extended detection window may reflect differences in histone turnover in distinct germline regions.

Our study also shows that the photoconversion methodology developed by others (Rosu and Cohen-Fix 2017) can be applied to provide a better tool for examining nuclear movement rate as opposed to Cy3/Cy5-dUTP or EdU labeling. This could be due to the fact that Cy3/Cy5-dUTP or EdU labels all S-phase replicating cells at the time of exposure and therefore nuclei throughout PMT are labeled (Crittenden et al. 2006; Morgan et al. 2010). This results in a wide spread of labeled nuclei in mitosis, which is likely contributed to by nuclei in the proximal PMT entering meiosis before those labeled in the distal PMT (Crittenden et al. 2006; Morgan et al. 2010). Moreover, as the starting point has to be in PMT, by the time nuclei reach LP, the rate of movement is affected by the sum of the small variations in prior zones, amplifying noise in the system. The Dendra2 photoconversion method allows a narrower population to be labeled (in our case, only 3 nuclear rows). Moreover, the conversion can be performed post-meiotic entry and in short proximity to where it is analyzed, reducing noise.

### Synthesis of models of dHJ/SDSA choice

In *C. elegans*, many more DSBs are generated than the single CO that is allowed per chromosome pair, so only a small subset of late DSBs become committed to the CO pathway, with the remaining DSBs directed to NCO repair by the SDSA stage (Saito and Colaiácovo 2017; Gartner and Engebrecht 2022). Our studies in *C. elegans* indicate that meiotic DSBs can be divided into 2 groups: early and late DSBs, based on their spatiotemporal formation, which influences the choice of repair pathway. The transition between the 2 groups of DSBs occurs in the middle of MP. It is hard to identify exactly where early DSBs start and where late DSBs end, but based on RAD-51 localization, early DSBs start to form in TZ, and the last late DSBs form in LP entry or shortly after. RAD-51 marks DSBs after they are processed to ssDNA. Therefore, the exact timing of DSB formation may be a bit earlier (but should show the same shift in timing for early and late DSBs). It is important to note that late and early DSBs each likely represent half of the DSBs formed. This estimation is based on the assumption that SPO-11::AID degradation is rapid (less than 30 min), similar to what was shown for other proteins using this system (Zhang et al. 2015). The presence of SPO-11 cannot be detected directly and is only inferred indirectly by RAD-51 foci disappearance. RAD-51 foci mostly disappear 4 h post-SPO-11 depletion, which includes the time required for SPO-11 degradation, RAD-51 homology search, and strand invasion (Hicks et al. 2022). However, most of these 4 h are likely taken by homology search and strand invasion, supported by the fact that the timing of RAD-51 foci disappearance is remarkably similar for loss of long-range resection mutants (Hicks et al. 2022) and recent kinetic analysis of RAD-51 (Hamrick et al. 2026). If SPO-11 degradation takes longer, that will lead to a small shift in the transition point between early-to-late DSBs (no more than 3 rows downstream), indicating that the population of late DSBs is slightly smaller than that of early DSBs.

Our studies do not contradict previous findings but are complementary to them. We show that DSBs that are generated from TZ to EP are only repaired as NCOs using SDSA. This may be an outcome of the lack of formation of bright COSA-1 foci in this stage, as shown by others (Yokoo et al. 2012). While both MSH-5 and COSA-1 form bright foci in LP, they do show dim foci earlier in meiosis (Woglar and Villeneuve 2018). The absence of bright COSA-1 foci in the early DSB region may prevent significant levels of MSH-4/5 stabilization on dHJ precursor sites, thus leading to the ejection of the invaded ssDNA and subsequent reannealing to the ssDNA on the other side of the break. DSBs formed in the late region (MP to LP entry) are present in nuclei that can localize COSA-1 to chromosomes (Yokoo et al. 2012), and therefore can produce dHJs and thereby COs. We propose that these late DSBs are subjected to CO interference, leading to a CO/NCO decision at the strand ejection step. We therefore propose that the signal for early-to-late DSB transition is governed by the recruitment of COSA-1 beyond a threshold of a “bright focus,” and could be influenced by known checkpoints activated in this stage, such as the CHK-2 checkpoint (Yokoo et al. 2012; Kim et al. 2015; Yang et al. 2024). Others have proposed that PCH-2 may be responsible for the signal (Patel et al. 2025). However, the effect of *pch-2* in the same system (wild-type background, *spo-11::AID*) is small and cannot be observed with COSA-1. While PCH-2 is required for the regulation of a subset of early DSBs, the picture is likely more complex, and the main player(s) remain unknown.

Our studies in *C. elegans* relate to a phenomenon that was observed in yeast: early “scout” DSBs, which are generated before the bulk of recombination initiates, have a different DSB repair fate (Joshi et al. 2015; Sandhu et al. 2020). These scout DSBs preferentially utilize sister chromatids, unlike late DSBs, which utilize homologous chromosomes. Thus, scout DSBs may be analogous to early DSBs, with the exception that early DSBs in *C. elegans* are repaired using homologous chromosomes and exclusively by SDSA, while scout DSBs in yeast use sister-chromatid exchange for DSB repair.

### Possible implication of the correlation between RAD-51 foci distribution and early-to-late DSB transition

In yeast, meiotic DSBs begin forming at meiotic entry (Padmore et al. 1991), but in other species, the precise timing of DSB formation remains unresolved. In yeast, *Arabidopsis*, and mouse, analysis of recombination intermediates shows a peak in zygotene to early pachytene (Moens et al. 2002; Kurzbauer et al. 2012; Borde and de Massy 2015). Our work, and that of others (*e.g.,* reviewed by Yu et al. 2016) in *C. elegans*, places the peak of RAD-51 foci numbers in MP, which is later compared to observations in other model systems. Since our current study indicates that early and late DSBs transition occurs at this later point, it suggests that in other species, this transition may be in zygotene, as opposed to MP, as found in worms. If so, an early transition may require higher resolution analysis for the parsing of early and late DSBs in other systems. Since this topic was not investigated outside yeast, we do not yet know how or if the timing of DSB formation affects their mode of repair in other systems. Our studies suggest a hypothesis framework for this analysis.

In summary, our findings support our previous studies (Hicks et al. 2022) that challenge the standard model of dHJ/SDSA choice in meiosis. We propose a more complex picture, in which DSBs are not homogeneous: early and late DSBs exhibit significant differences in repair outcomes.

## Supplementary Material

jkag130_Supplementary_Data

## Data Availability

Strains are available upon request. The authors affirm that all data necessary for confirming the conclusions of the article are present within the article, figures, and tables, including raw data that can be found in the Supplementary file “Supplementary Information—Raw Data.” Supplemental material available at G3 online.
