# Supplementary material for: The mid-pachytene transition between early and late double-strand breaks in C. elegans meiosis is conserved throughout early adulthood and coincides with a peak in recombination intermediates
Source: G3 (Bethesda). 2026 May 14;16(8):jkag130. doi: 10.1093/g3journal/jkag130 (PMC13439936; doi:10.1093/g3journal/jkag130)
Supplement: Supplementary file 1 [file jkag130_supplementary_data.zip › Supplementary_Figure_Legends_and_Tables_G3-2026-406769.docx]

**The mid-pachytene transition between early and late double-strand breaks in *C. elegans* meiosis is conserved throughout early adulthood and coincides with a peak in recombination intermediates**

Hui Tian, Korie Campbell, and Sarit Smolikove

**Supplementary Figure Legends and Tables**

**Supplementary Fig. 1. Nuclear movement gradually slows down through the germline at the 12h post-L4 time point.**

a) Schematic representation of the experimental setup of photoconversion. Three experiments were conducted investigating three different germline regions. Red boxes represent the photoconverted region and (on the left) the imaged region (on the right). b) Representative images of gonad ~12 hours after photoconversion at TZ (green for Dendra2::H2B, red for Photoconverted Dendra2::H2B). Scale bar, 10 μm. c) Quantification of nuclear movement rate by rows when photoconverted at the different positions as shown in a. d) Schematic representation of nuclear movement rate in the gonad. e) Quantification of width of row at different meiotic stages in gonad. f) Quantification of nuclear movement rate by distance per hour at different meiotic stages in gonad. All analyses were performed in at least five gonads and in biological replicates. Data are shown as mean ± SEM. Asterisks indicate statistical significance calculated by Mann-Whitney test (∗∗p < 0.01, ns - not significant).

**Supplementary Fig. 2. Quantification of RAD-51 foci in *spo-11::AID; ollas::cosa-1* worms by zones.**

a and b) Quantification of RAD-51 foci *in spo-11::AID; ollas::cosa-1* worms by zones at the indicated time points (a: L4, 8 hours post L4, 12 hours post L4; b: 24 hours post L4, 36 hours post L4, 48 hours post L4). All analyses were performed in three gonads per time point. Data are shown as mean ± SEM. For L4, 8 hours post L4, 12 hours post L4, and 24 hours post L4, zone 1 and 2 is PMT, zone 3 is TZ, zone 4 - zone 7 is pachytene. For 36 hours post L4, 48 hours post L4, zone 1 and 2 is PMT, zone 3 is TZ, zone 4 - zone 9 is pachytene. No statistics were applied, because zones are different between different time points.

**Supplementary Fig. 3. Inhibition of SPO-11 function in LP prevents the formation of crossovers.**

a) Quantification of COSA-1 foci per nucleus in LP in different time points during development of *spo-11::AID; ollas::cosa-1* worms. LP: late pachytene. b) Representative images of DAPI bodies from three representative time points during development of *spo-11::AID; ollas::cosa-1* worms. Scale bar, 1 μm. c and d) Quantification of DAPI bodies in different time points during development of *spo-11::AID; ollas::cosa-1* worms. All analyses were performed in at least three gonads. Data are shown as mean ± SEM.

**Supplementary Tables**

**Supplementary Table 1. The peak of RAD-51 foci per nucleus.** This table displays left row, right row, middle row and total rows of the pachytene region at 6 time points. “Left row” and “Right row” respectively represent the two left rows showing a significant increase and two right rows showing a significant decrease in RAD-51 foci per nucleus. “Middle row” represents the middle of the second left row and the first right row. “Total rows” is the average of total pachytene rows. Asterisks indicate statistical significance in average number of foci in the RAD-51 peak region, as calculated by Mann-Whitney test (*p < 0.05, ns - not significant)

| Hours post-L4 | Left  Row | Right  Row | Middle  Row | Total rows (of average) | Comparing with 24 hours post L4 |
| --- | --- | --- | --- | --- | --- |
| 0 | 5 vs. 7 | 14 vs. 18 | 10.5 | 20 | * |
| 8 | 7 vs. 10 | 18 vs. 22 | 14 | 26 | ns |
| 12 | 10 vs. 12 | 22 vs 23 | 17 | 34 | ns |
| 24 | 15 vs. 17 | 26 vs. 30 | 21.5 | 44 |  |
| 36 | 13 vs. 15 | 32 vs. 35 | 23.5 | 52 | * |
| 48 | 15 vs. 18 | 40 vs. 42 | 29 | 60 | * |

**Supplementary Table 2. Average number of COSA-1 foci in *spo-11::AID; OLLAS::cosa-1*** **strain.** This table shows the average number of COSA-1 foci per nucleus in LP, along with SD and SEM. L4 stage worms were grown on NGM plates for the duration indicated in the above table, then transferred to auxin plates for continued growth to remove SPO-11 at a specific time window. “Age at time of analysis” represents the total time the L4 worms were grown on NGM/auxin plates for this experiment. Asterisks indicate statistical significance calculated by Mann-Whitney test (*p < 0.05, ∗∗p < 0.01, ****p < 0.0001, ns - not significant)

| Hours from L4  on  NGM | Exposure hours  on  auxin | Age at time of analysis | Average number of COSA-1 foci | SD | SEM | Comparing to time point before | Comparing with 18 hours auxin exposure |
| --- | --- | --- | --- | --- | --- | --- | --- |
| 24 | 0 | 24 | 5.9 | 0.38 | 0.05 |  | **** |
| 0 | 8 | 8 | 4.5 | 2.32 | 0.31 |  | **** |
| 0 | 12 | 12 | 3.7 | 2.21 | 0.29 | * | **** |
| 0 | 18 | 18 | 0.4 | 0.66 | 0.08 | **** |  |
| 0 | 24 | 24 | 0.2 | 0.43 | 0.06 | ns | ns |
| 8 | 12 | 20 | 5.4 | 1.43 | 0.20 |  | **** |
| 8 | 15 | 23 | 2.8 | 2.15 | 0.27 | **** | **** |
| 8 | 18 | 26 | 0.3 | 0.64 | 0.08 | **** | ns |
| 10 | 20 | 30 | 1.1 | 1.52 | 0.22 |  | ** |
| 10 | 22 | 32 | 0.5 | 0.63 | 0.08 | * | ns |
| 12 | 18 | 30 | 4.6 | 1.65 | 0.23 |  | **** |
| 12 | 21 | 33 | 2.8 | 1.78 | 0.27 | **** | **** |
| 12 | 24 | 36 | 0.6 | 0.91 | 0.12 | **** | ns |
| 20 | 28 | 48 | 1.2 | 1.96 | 0.26 |  | * |
| 20 | 30 | 50 | 0.3 | 0.51 | 0.07 | * | ns |
| 24 | 24 | 48 | 3.9 | 2.36 | 0.33 |  | **** |
| 24 | 30 | 54 | 1.1 | 1.65 | 0.20 | **** | * |
| 24 | 32 | 56 | 0.5 | 0.73 | 0.09 | ns | ns |
| 30 | 34 | 64 | 1.3 | 1.50 | 0.23 |  | **** |
| 30 | 36 | 66 | 0.4 | 0.50 | 0.09 | ** | ns |
| 36 | 24 | 60 | 5.1 | 1.48 | 0.19 |  | **** |
| 36 | 36 | 72 | 2.0 | 1.98 | 0.26 | **** | **** |
| 36 | 40 | 76 | 0.2 | 0.48 | 0.06 | **** | ns |

**Supplementary Table 3. Average number of DAPI bodies in *spo-11::AID; OLLAS::cosa-1* strain.** This table shows the average number of DAPI bodies in D-1 oocytes. L4 stage worms were grown on NGM plates for the duration indicated in the above table. They were transferred to auxin plates for continued growth, before being moved back to NGM plates, which indicated that SPO-11 was removed during a specific time window. “Age at time of analysis” represents the total time that L4 worms were grown on NGM/auxin plates for this experiment.

| Hours from L4  on  NGM | Exposure hours  on  auxin | Off auxin  (back on NGM) | Age at time of analysis | Average number of DAPI bodies |
| --- | --- | --- | --- | --- |
| 24 | 0 | 0 | 24 | 6 |
| 0 | 24 | 0 | 24 | 12 |
| 8 | 12 | 28 | 48 | 10.32 |
| 8 | 12 | 32 | 52 | 11.42 |
| 8 | 16 | 24 | 48 | 10.72 |
| 12 | 12 | 24 | 48 | 7.14 |
| 12 | 12 | 28 | 52 | 8.33 |
| 12 | 24 | 8 | 44 | 6.70 |
| 12 | 24 | 12 | 48 | 11.28 |
| 12 | 24 | 18 | 54 | 11.88 |
| 12 | 24 | 24 | 60 | 11.93 |
| 12 | 24 | 30 | 66 | 11.21 |
| 12 | 24 | 36 | 72 | 9.47 |
